# Identification of SARS-CoV-2–induced pathways reveals drug repurposing strategies

**DOI:** 10.1126/sciadv.abh3032

**Published:** 2021-06-30

**Authors:** Namshik Han, Woochang Hwang, Konstantinos Tzelepis, Patrick Schmerer, Eliza Yankova, Méabh MacMahon, Winnie Lei, Nicholas M. Katritsis, Anika Liu, Ulrike Felgenhauer, Alison Schuldt, Rebecca Harris, Kathryn Chapman, Frank McCaughan, Friedemann Weber, Tony Kouzarides

**Affiliations:** 1 Milner Therapeutics Institute, University of Cambridge, Cambridge, UK.; 2 Institute for Virology, FB10-Veterinary Medicine, Justus-Liebig University, Gießen 35392, Germany.; 3 Centre for Therapeutics Discovery, LifeArc, Stevenage, UK.; 4 Department of Surgery, University of Cambridge, Cambridge, UK.; 5 Department of Chemical Engineering and Biotechnology, University of Cambridge, Cambridge, UK.; 6 Department of Chemistry, University of Cambridge, Cambridge, UK.; 7 Data and Computational Sciences, GSK, London, UK.; 8 Department of Medicine, University of Cambridge, Cambridge, UK.; 9 The Gurdon Institute and Department of Pathology, University of Cambridge, Cambridge, UK.

## Abstract

The global outbreak of severe acute respiratory syndrome coronavirus 2 (SARS-CoV-2) necessitates the rapid development of new therapies against coronavirus disease 2019 (COVID-19) infection. Here, we present the identification of 200 approved drugs, appropriate for repurposing against COVID-19. We constructed a SARS-CoV-2–induced protein network, based on disease signatures defined by COVID-19 multiomics datasets, and cross-examined these pathways against approved drugs. This analysis identified 200 drugs predicted to target SARS-CoV-2–induced pathways, 40 of which are already in COVID-19 clinical trials, testifying to the validity of the approach. Using artificial neural network analysis, we classified these 200 drugs into nine distinct pathways, within two overarching mechanisms of action (MoAs): viral replication (126) and immune response (74). Two drugs (proguanil and sulfasalazine) implicated in viral replication were shown to inhibit replication in cell assays. This unbiased and validated analysis opens new avenues for the rapid repurposing of approved drugs into clinical trials.

## Teaser

Data-driven computational analysis and simulation identified drug repurposing opportunities for COVID-19.

## INTRODUCTION

**Fig. 1:**
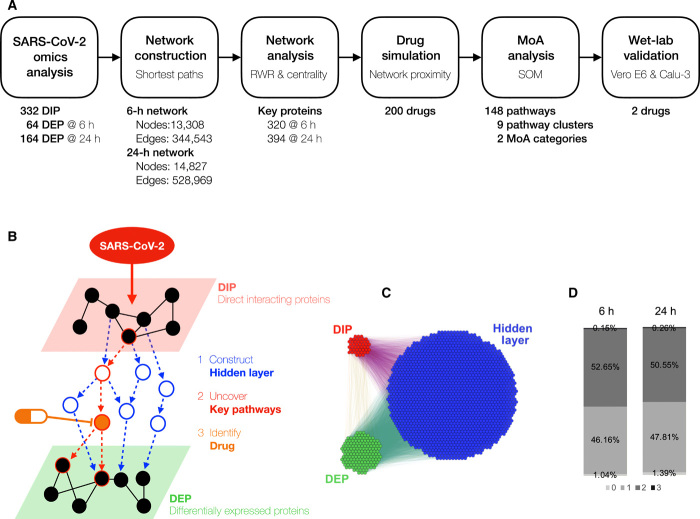
Construction of a SIP network. (**A**) Overview and workflow of the in silico drug repurposing pipeline. (**B**) Schematic depicts our strategy of constructing a SIP hidden network through data integration and network construction of DIPs and DEPs, followed by identification of drugs that target key pathways in this network. (**C**) The SARS-CoV-2 Orf8 subnetwork shows the extent of the hidden layer that is revealed through the network analysis. (**D**) Percentage of the shortest paths between the DIP and DEP that are via zero to three proteins at 6 hours versus 24 hours.

To date, most small-molecule and antibody approaches for treating severe acute respiratory syndrome coronavirus 2 (SARS-CoV-2)–related pathology are rightly rooted in repurposing and are focused on several key virus or host targets or on pathways as points for therapeutic intervention and treatment. This has been underpinned by the unprecedented pace of scientific research to uncover the molecular bases of virus structure and the mechanisms by which it gains access to cells before replication and release of new virus particles. The emergence of global proteomic datasets is now propelling our understanding of the mechanisms through which the virus interacts with host cell proteins, determining the directly interacting proteins (DIPs) (*1*) and differentially expressed proteins (DEPs) (*2*). Such interactome outputs and related efforts in transcriptomics (*3*) have begun to provide detailed information on possible individual targets and pathways against which currently available drugs can be tested for potential coronavirus disease 2019 (COVID-19) repurposing. Systematic analyses of these datasets will direct further research toward likely points of successful therapeutic intervention. In this study, we have applied the power of bespoke computational biology and machine learning approaches to dissect these datasets and construct an agnostic network for SARS-CoV-2–induced pathways, uncovering novel targets and potential repurposing strategies (Fig. 1A). We have focused our study on host-directed therapy, an emerging and complementary approach to virus-targeting drugs, that interferes with signaling mechanisms in the host cell to effectively inhibit the productivity of viral replication (*4*).

## RESULTS

### Construction of a SARS-CoV-2–induced protein network

To determine the disease mechanisms underlying a SARS-CoV-2 infection, we undertook a comprehensive analysis of the protein pathways implicated in COVID-19, using computational biology workflows for data integration and network construction. To this end, we hypothesized that DIPs are the “cause” and DEPs are the “consequence” of SARS-CoV-2 infection. We then constructed a SARS-CoV-2–induced protein (SIP) network in which all DIP and DEP combinations are connected to understand how the chains of the cause and consequences are connected (Fig. 1B). We identified all the possible shortest paths between 332 DIP and 64 DEP combinations in the SIP network at 6 hours and between 332 DIP and 164 DEP combinations in the SIP network at 24 hours using the human STRING database (*5*). In our SIP network, there are three layers: the DIP, the DEP, and the hidden layer between the two. Our analysis of the DEP data identified DIPs at 6 and 24 hours after infection; hence, we constructed the SIP network for these two time points. There are 13,308 proteins and 344,543 interactions in the 6-hour network and 14,827 proteins and 528,969 interactions in the 24-hour network [fig. S1 shows the entire SIP network at the two time points; Fig. 1C shows a subnetwork of SARS-CoV-2 Orf8 (Open reading frame 8) at 24 hours]. Almost 99% of the DIP-to-DEP paths in both networks are via more than one protein (Fig. 1D), and there is only a 2% overlap between DIP and DEP. It suggests that the “hidden layer” that we have constructed in our network is central to understanding the pathways that connect DIPs and DEPs and allows us to discover novel relationships by integrating the datasets.

### The SIP network can be interrogated to reveal key proteins and disease pathways

**Fig. 2:**
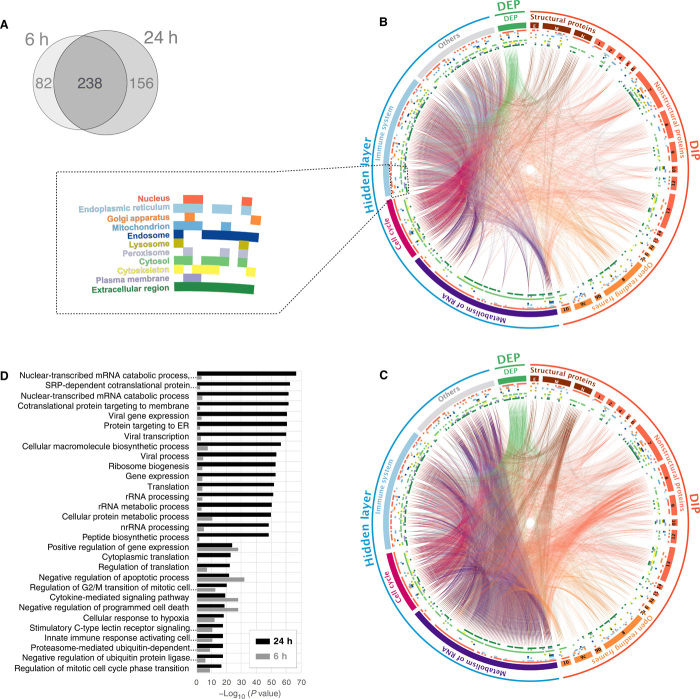
SARS-CoV-2 viral protein subnetwork analysis shows an enrichment of viral replication pathways. (**A**) Venn diagram of key proteins in 6- and 24-hour SIP networks. (**B**) A circos plot depicting interactions between DIPs and DEPs revealed through the SIP network at 6 hours after infection. DIPs were subdivided into the genomic organization of SARS-CoV-2. Proteins in the hidden layer were also subdivided into major pathways. Inner colored circles demonstrate the subcellular localization of the proteins, and details are shown in the dotted box. The colored lines show PPI. (**C**) Twenty-four hours after infection. (**D**) Top 30 enriched GO terms of the key proteins in the SIP network at 24 hours (black). The enrichment *P* values of 30 terms at 6 hours are also shown as a control (gray).

In protein-protein interaction (PPI) networks, proteins that are central to a pathway are desirable as druggable targets because they may have a greater impact on pathway function. To identify key proteins and disease pathways in the SIP network, we applied multiple network algorithms [including eigenvector centrality, degree centrality, betweenness centrality, and random walk with restart (RWR)]. To identify statistically significant proteins, we performed 1000 permutation tests for each network algorithm and selected proteins with empirical *P* values less than 0.01. The proteins selected by each network algorithm were merged and considered as key proteins (see Materials and Methods). This revealed 320 proteins at 6 hours and 394 proteins at 24 hours, of which 238 (50% of 476 proteins) proteins were in common (Fig. 2A). More than half of the proteins identified as significant at both time points were in the hidden layer: 170 (53%) and 202 (51%), respectively (fig. S2, A and B). We then asked whether these proteins were also biologically relevant to the disease symptoms caused by COVID-19. A disease enrichment analysis on the proteins showed that the top 10 enriched diseases for these proteins at both time points are diseases that are potentially relevant for COVID-19 pathogenesis, including lung disease (*6*, *7*), hypertension (*6*, *7*), and hyperglycemia (table S1) (*6*). To uncover potential biological functions of the important proteins at 6 hours, 24 hours, and both time points, we tested for enrichment of disease (*8*) and Gene Ontology (GO biological process) terms to characterize the key proteins in the SIP network. For proteins at 6 hours and proteins that are common to both time points, the pathways were related to the immune system and virus replication (VR) (fig. S3, A and B). In contrast, the pathways that were relevant for the proteins at 24 hours were primarily related to VR (fig. S3C). In this way, we established a COVID-19 SIP network that allows investigation of disease pathways that are pertinent to SARS-CoV-2 infection.

### SARS-CoV-2 viral protein subnetwork analysis demonstrates an enrichment of pathways related to viral replication

SARS-CoV-2 has a large RNA viral genome (~30,000 nucleotides) with subgenomic structures that produce 29 viral proteins (4 structural proteins, 16 nonstructural proteins, and 9 accessory factors of the virus genome). To understand the disease mechanism of COVID-19, we investigated the subnetwork for each of these viral proteins and asked which biological processes these are implicated in. We analyzed several parameters for key proteins in each subnetwork: (i) the differences between the 6- and 24-hour time points (Fig. 2, B and C, and table S2); (ii) the subcellular localization of the key proteins (table S3); and (iii) the biological processes that the key proteins act in (table S4).

First, we found a significantly increased number of interactions with RNA metabolism at 24 hours (1504 interactions at 6 hours but 6794 at 24 hours with a *P* value of 2.2 × 10^−16^; Fig. 2D and table S2). We observe that the viral proteins N (Nucleocapsid), Nsp 8 (Nonstructural protein 8), and Orf8 and Orf10 of SARS-CoV-2 interact with ribosomal proteins in the hidden layer of our SIP network, indicating that they may have a possible influence on RNA metabolism (Fig. 2, B and C, and table S2). The N and Nsp 8 proteins are known to drive viral replication (*1*). Orf8 and Orf10 are the only two proteins of SARS-CoV-2 that are distinct from other coronaviruses (*9*). We also observed that Orf8-interacting DIPs were enriched in the endoplasmic reticulum (ER) (Fig. 2, B and C, and table S3), which may be significant as the ER is the intracellular niche for viral replication and assembly (*10*). Of the 28 proteins that SARS-CoV-2 Orf8 directly interacts with, 13 (46.43%, *P* value: 3.18 × 10^−6^) are localized in the ER, compared with only 11.84% (36) of all other DIPs (304) being localized in ER.

We then sought the most relevant biological pathways—immune system and viral replication—that have previously been described for SARS-CoV-2 (*11*) at the highest hierarchical level in the Reactome pathway database. The “immune system” (*P* value: 9.57 × 10^−18^) (*12*) was identified for the immune response (IR). The “metabolism of RNA” (*P* value: 5.37 × 10^−45^) (*12*, *13*) and “cell cycle” (*P* value: 1.73 × 10^−16^) (*14*) were found for viral replication. The key proteins belonging to these three pathways were assigned to the three subgroups (purple, metabolism of RNA; red, cell cycle; and light blue, immune system) under the hidden layer in Fig. 2 (B and C). The key proteins that did not belong to any of the three pathways were assigned to “others.” There were 54 key proteins in the hidden layer that did not have strong enrichment in the Reactome pathways (other) but that still actively interacted with metabolism of RNA proteins at 24 hours (Fig. 2C and tables S2 and S4). Further study on the other proteins found individual links to RNA binding (ATP5A1, MRTO4, and NHP2L1), host-virus interaction (ACE2, CXCR4, DERL1, GNB2L1, HSPD1, KDR, KRT18, SIRT1, and TMPRSS2), histones (H2AFZ, HIST2H3PS2, and WDTC1), viral mRNA translation (MRPS7), and ER-associated responses (ATF4, CFTR, DERL1, and INS).

We next confirmed statistically that virus-related pathways are enriched in the top 30 enriched GO terms (*P* value less than 4.64 × 10^−17^) of 976 enriched GO terms (*P* value less than 0.05) as well as RNA- and ER-related processes (Fig. 2D; see fig. S4, A and B, for the top 150 terms and table S4 for all enriched GO terms). The differences between the two time points were also confirmed. In summary, our pattern analysis in the SARS-CoV-2 viral protein subnetworks revealed which biological pathways change significantly during the course of infection, with prominent increases in proteins involved in VR by 24 hours (Fig. 2D).

### An in silico network proximity analysis of drug-target relationships identifies drug candidates

Having identified key SIP proteins, we were motivated to identify approved drugs that bound a significant number of these host proteins and which might therefore have stronger effects in blocking SARS-CoV-2–induced changes. We conducted an in silico network-based proximity measure analysis (*15*) on the key proteins of the SIP network at 6 and 24 hours after infection. We collected 1917 approved drugs from publicly available databases [ChEMBL (*16*) and DrugBank (*17*); table S5]. This virtual screening identified 200 drugs (table S6) that are predicted to target the key proteins of the SIP network, of which 99 (49.5%) were specific to the 6-hour time point, 14 (7%) were specific to the 24-hour time point, and 87 (43.5%) were common to both time points. We then checked the Anatomical Therapeutic Chemical code (available for 180 drugs only) to determine the therapeutic areas for which specific drugs have been developed. The top clinical areas against which these approved drugs are used for were cancer, sex hormone signaling, diabetes, immune system, bacterial disease, and inflammatory/rheumatic disease (fig. S5). A total of 35% of the 200 drugs have been tested in phase 2 or 3 clinical trials for infectious diseases, and half of these were HIV trials; furthermore, 16% of drugs have been tested in trials for inflammatory and 10% in respiratory disease.

Among the 200 identified drugs, 40 (20%) are now in COVID-19 clinical trials (tables S6 and S7) (*18*). To determine the significance of this finding, we asked what the likelihood would be of this number of drugs being identified as hits by chance. We found that, by comparison, only 13% of the approved drugs (249 of 1917) were in the COVID-19 clinical trials (*18*). A hypergeometric test for the probability of 20% of our 200 drugs being in clinical trials returned a *P* value of 3.59 × 10^−3^, demonstrating the utility of our integrated computational approaches for prioritizing compounds. Of the 200 drugs identified, a further total of 30 drugs have also been reported as being potential candidates against COVID-19 (*19*–*24*). Thus, network-based proximity analysis has revealed 70 drugs in total that are either in COVID-19 clinical trials or being considered as potential drug candidates in preclinical studies, supporting the strength of our approach. In this way, our analysis has identified a total of 130 drugs that could provide novel opportunities for repurposing as COVID-19 therapeutics. The full list of 200 approved drugs along with their detailed information is shown in table S6.

### Artificial neural network analysis uncovers drug mechanisms of action

We next wanted to establish the mechanism of action (MoA) underlying the 200 identified drugs. In particular, we wanted to cluster the pathways and mechanisms to better evaluate their potential effect and utility. An initial pathway enrichment test performed on the proteins that are targeted by the 200 drugs identified a set of 148 key pathways (see Materials and Methods). We then calculated the precision and recall of the enrichment test to produce an F1 score that is the measure of the enrichment accuracy (see Materials and Methods). The F1 scores were calculated per drug-pathway association; in this way, we generated an F1 score matrix (for the 200 drugs and 148 key pathways; table S8). To investigate the MoA (that is, the profile of pathways in which drug targets are significantly enriched) for the 200 drugs in the context of COVID-19, we used a self-organizing map (SOM), a type of artificial neural network, to analyze the relationship between the 200 drugs and the 148 key pathways (termed as drug-pathway association).

**Fig. 3:**
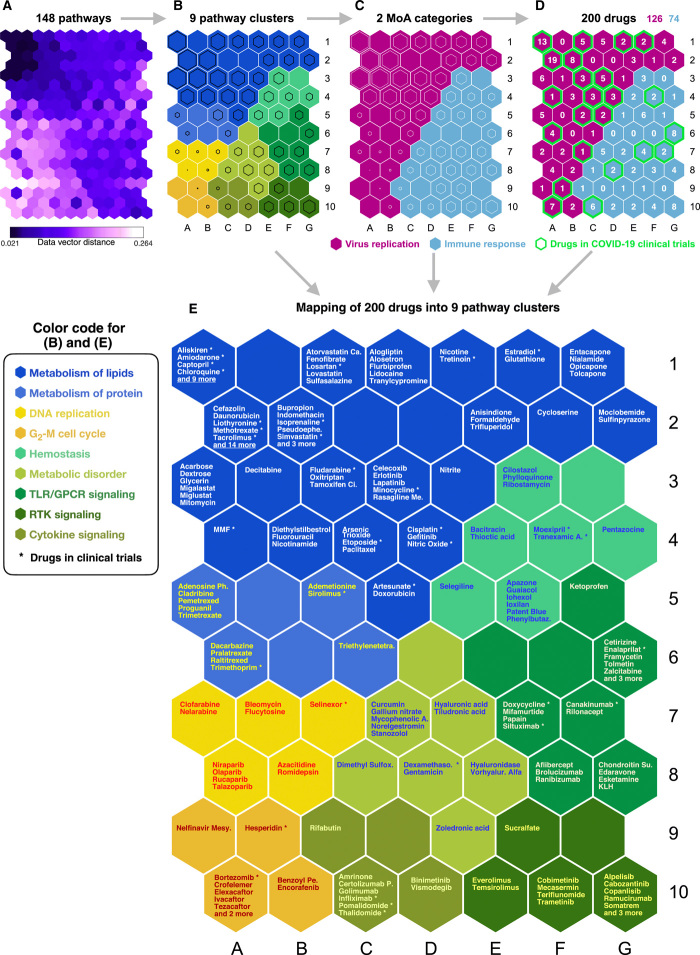
Machine learning predicts MoAs for the 200 drug repurposing candidates. (**A**) U-matrix is shown of the trained unsupervised SOM used to analyze the relationship between the 200 drugs and the 148 key pathways. This contains the distance (similarity) between the neighboring SOM neurons (pathways) and shows data density (drug-pathway association scores) in input space. Each hexagon is colored according to distance between corresponding data vectors of neighbor neurons, with low-distance areas (dark purple) indicating high data density (clusters). Each smaller hexagon on the U-matrix (A) indicates the data vector distance between larger hexagons in the SOM cluster arrangements (B to E). Thus, a smaller hexagon on the U-matrix corresponds to every adjacent larger hexagon on the SOM cluster arrangements (B to E). (**B**) The selected clustering arrangement was based on the U-matrix and DBI to separate the 148 key pathways into nine clusters. The names of nine clusters are shown in the figure. Clusters of each SOM neuron are distinguishable by color. The size of the black hexagon in each neuron indicates distance. Larger hexagons have a low distance to neighboring neurons, hence forming a stronger cluster with neighbors. (**C**) Two MoA categories were identified on the basis of the pathway clustering and the drug mapping. (**D**) Mapping of the 200 identified drugs to each neuron (pathway) based on matching rates and inspection of examples from each cluster. (**E**) SOM component map shows mapping results of the 200 drugs into nine pathway clusters. The names of the nine clusters are shown in the figure, and the drugs with asterisk are already in COVID-19 clinical trials.

First, to characterize each of the 148 key pathways, the unsupervised training of SOM with the F1 score matrix generated 148 SOM component plane heatmaps (fig. S6). The SOM successfully predicted highly correlated pathways, although only the F1 scores and no prior biological knowledge of the 148 key pathway or the 200 drugs were used in the SOM training. Each heatmap represents the intensity patterns of a pathway, and each hexagon in the heatmap is a unique neuron or “node” of the SOM artificial neural network. To allow direct comparison between heatmaps (pathways), the hexagons (neurons) have the same position across all heatmaps. In this way, a group of pathways are correlated if their heatmaps are visually similar. For instance, three heatmaps at the grid positions of A7, B7, and C7 in fig. S6 are visually similar. The three heatmaps represent pathways for “G_1_-S transition,” “G_2_-M checkpoints,” and “G_2_-M transition”; thus, they are biologically correlated in cell cycle. To summarize the correlation of 148 heatmaps, the unified distance matrix (U-matrix) between the neighbor neurons was also calculated and presented in different colored hexagons, which illustrates the probability density distribution of data vectors (drug-pathway association score) (Fig. 3A) (*25*).

Next, the 148 key pathways were separated into nine clusters by a k-means clustering algorithm with Davies-Bouldin index (DBI). The nine clusters were “metabolism of lipids,” “metabolism of protein,” “DNA replication,” “G_2_ or M cell cycle,” “hemostasis,” “metabolic disorder,” “Toll-like receptor (TLR) or G protein–coupled receptor (GPCR) signaling,” “receptor tyrosine kinase (RTK) signaling,” and “cytokine signaling” (shown in different colors in Fig. 3B). To determine the optimal number of clusters, we calculated the DBI based on the U-matrix. The lowest DBI value occurs at nine clusters (fig. S7); thus, we decided to separate the 148 key pathways into nine pathway clusters. The size of the black hexagon in each colored hexagon indicates distance to its neighbor hexagon; thus, a larger black hexagon indicates more correlation with its neighbor hexagons.

The nine pathway clusters were then mapped into potentially important MoA categories for SARS-CoV-2 infection by pathway analysis (table S9). To identify these categories, we first searched the COVID-19–related literature and determined that there are mainly “two broad categories” of disease mechanism reported: (i) IR and (ii) viral replication (*11*). We then mapped the nine pathway clusters based on two factors: (i) biological supporting evidence from the literature and (ii) computationally inferred evidence from SOM clustering arrangements. The detailed source of the biological supporting evidence is shown in table S9. The computationally inferred evidence was provided by the SOM clustering arrangements between the nine pathway clusters (Fig. 3B). For instance, RTK signaling is closely positioned by two hallmark immune system pathways (cytokine signaling and TLR/GPCR signaling) on the SOM clustering arrangements (Fig. 3B). Thus, RTK signaling was predicted to have a high probability of having a role in the IR. The mapping revealed two MoA categories that could explain the mechanisms of the 200 identified drugs. The two MoA categories were VR and IR (Fig. 3C). For instance, 47 pathways among the 148 key pathways are related to metabolism of lipids that plays a key role at various stages in viral replication, including entry, uncoating, genome replication, assembly, and release (*26*). There are 18 pathways related to DNA replication, and it is known that intermediate and late viral mRNAs concentrate in DNA replication factories (*27*). We also found seven cytokine signaling pathways that regulate the IR (*28*). The entire mapping results and supporting evidence are provided in table S9.

Last, the SOM mapped the 200 drugs into each neuron and hence the key pathways (the number of drugs per neuron is shown in Fig. 3D, and drug names are shown in Fig. 3E). Notably, 30 of the 40 drugs that are in COVID-19 clinical trials (*18*) were in the VR MoA category, while only 10 drugs were in the IR (Fig. 3D). We then identified mechanistic roles and connections for the 200 drugs and their target proteins and mapped the drugs into nine pathway clusters (Fig. 3E). A more extensive analysis of information about each drug is given in table S6.

### Proguanil and sulfasalazine reduce SARS-CoV-2 replication

We next sought to identify the precise proteins within the SIP network that are targeted by each of the 200 drugs. We found that, of the 1573 proteins targeted by the 200 drugs, most (66%) are targeted by a single drug (fig. S8A). However, there are 30 proteins (0.19%) that are targeted by eight or more drugs (*P* value less than 0.00757; fig. S8A). To establish whether there is a pathway relationship between these 30 proteins, we interrogated their molecular function. Figure S8B shows that the most enriched categories of function for these proteins were heme, microsome, oxidoreductase, and monooxygenase, all of which are related to nicotinamide adenine dinucleotide phosphate (NADP) and nitric oxide (NO) synthesis. As NO is important for viral synthesis (and because NADP affects NO production), this could provide a potential mechanism by which these drugs might alter viral infection (*29*–*31*). On the basis of these findings, we decided to validate, in cellular assays, five drugs (ademetionine, alogliptin, flucytosine, proguanil, and sulfasalazine) with good safety profiles that are functioning within this pathway. Compounds targeting the same pathway but with serious safety issues were not progressed for cellular validation.

**Fig. 4:**
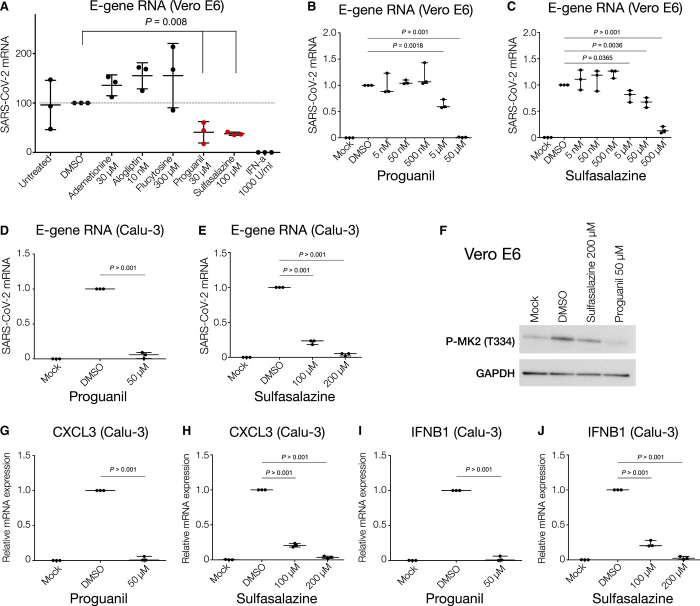
Proguanil and sulfasalazine reduce SARS-CoV-2 replication and p38/MAPK signaling activity. (**A**) RT-qPCR analysis of the indicated mRNA (envelope, E-protein) from Vero E6 cells pretreated with the indicated drugs and concentrations for 3 hours before infection with SARS-CoV-2 for 24 hours. Student’s *t* test. Means + SD of three independent replicates are shown. (**B** and **C**) RT-qPCR analysis of indicated mRNA (envelope, E-protein) from Vero E6 cells pretreated with proguanil or sulfasalazine at indicated concentrations for 3 hours before infection with SARS-CoV-2 for 24 hours. Student’s *t* test. Means + SD of three independent replicates are shown. (**D** and **E**) RT-qPCR analysis of indicated mRNA (envelope, E-protein) from Calu-3 cells pretreated with proguanil or sulfasalazine at indicated concentrations for 3 hours before infection with SARS-CoV-2 for 24 hours. Student’s *t* test. Means + SD of three independent replicates are shown. (**F**) Western blot analysis of phosphorylated MAPKAPK2 (Thr^334^) in mock-, DMSO-, sulfasalazine-, or proguanil-treated Vero E6 cells at indicated concentrations for 3 hours before infection with SARS-CoV-2 for 24 hours. (**G** to **J**) RT-qPCR analysis of the indicated mRNAs from Calu-3 cells pretreated with proguanil or sulfasalazine at indicated concentrations for 3 hours before infection with SARS-CoV-2 for 24 hours. Student’s *t* test. Means + SD of three independent replicates are shown.

To assess whether these five drugs are able to reduce SARS-CoV-2 infection, we performed an initial screening using the monkey Vero E6 cell line, where we observed that two of the five drugs, namely proguanil and sulfasalazine, showed significant antiviral effects without any noticeable cellular toxicity at the indicated doses (Fig. 4A and fig. S9A). We then focused on these two drugs, expanding our validation using the human Calu-3 cell line (in addition to Vero E6 cells). Treatment of Vero E6 and Calu-3 cells with proguanil and sulfasalazine illustrated strong anti–SARS-CoV-2 effects (represented by reductions of the envelope and nucleocapsid gene RNAs) in a dose-dependent manner, mirroring the results of the initial screen (Fig. 4, B to E, and fig. S9, B to E). No significant effect on cellular viability was observed at any tested dose (fig. S9, F to H). The effective concentration of sulfasalazine is comparable to maximal plasma concentrations achieved routinely in patients with rheumatoid arthritis or inflammatory bowel disease (*32*).

To further demonstrate the anti–SARS-CoV-2 impact of these two drugs, we examined the status of recently found intracellular pathways directly associated with SARS-CoV-2 infection and cytokine production (*33*). Treatment with either proguanil or sulfasalazine significantly reduced the phosphorylation of MAPKAPK2 (p-MK2 and T334) (Fig. 4F), an important component of the p38/mitogen-activated protein kinase (MAPK) signaling pathway, which has been shown to be activated via SARS-CoV-2 infection and stimulate cytokine response (*33*). Treatment of Calu-3 and Vero E6 cell lines with proguanil and sulfasalazine led to a significant down-regulation of the mRNA of key cytokines (Fig. 4, G to J, and fig. S10), which are dictated by the p38/MAPK signaling pathway and shown to become elevated during SARS-CoV-2 infection and replication (*CXCL3*, *IFNB1*, and *TNF-A*) (*33*). Hence, the above results solidify the promising anti–SARS-CoV-2 effects of the two drugs, both at the viral and the molecular level.

## DISCUSSION

Here, we have used a series of computational approaches—including bespoke methods for data integration, network analysis, computer simulation, and machine learning—to identify novel SARS-CoV-2–induced pathways that could be targeted therapeutically by repurposing existing and approved drugs (Fig. 1A). Although network analysis is increasingly being used for the analysis of genetic datasets to uncover disease signatures (*34*), a few key aspects of our approach were essential in uncovering these new targets, including agnostic construction of the SIP network and application of novel algorithms (previously used in other industries including social media). In addition, the use of artificial neural networks to understand systematically the MoA for the drugs was vital to this investigation.

Our analysis identifies 200 approved drugs, along with their MoA, that may be effective against COVID-19 (table S6). We are confident that these drugs have a potential for repurposing for COVID-19, since 40 of the 200 drugs have already entered clinical trials, testifying to the discovery value of our approach. An important part of our analysis is the use of already approved drugs. This allows for the rapid advancement of the most promising of the 160 drugs that are not yet in clinical trials.

**Fig. 5:**
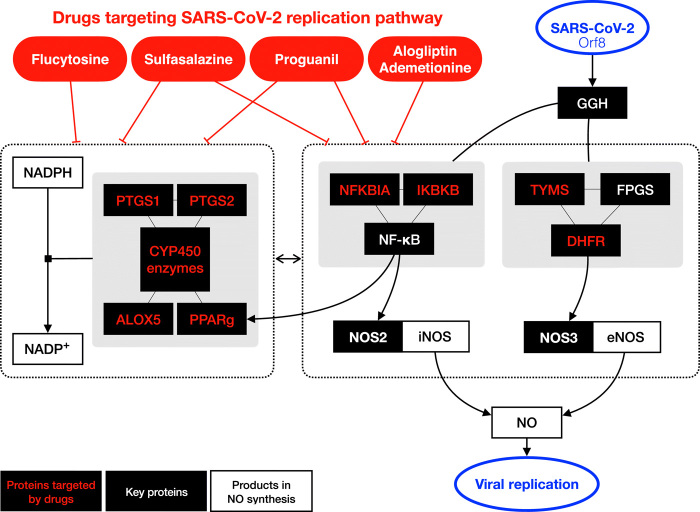
Schematics depicting the pathways mediating NO production that are targeted by the five tested drugs. The black boxes indicate key proteins in SIP network, and those targeted by the five drugs are highlighted in red color. Sulfasalazine and proguanil target proteins in both pathways that directly and indirectly (via NADP production) affect NO production (*58*–*61*).

We identify two drugs, sulfasalazine and proguanil, that can reduce SARS-CoV-2 viral replication in cellular assays, raising the exciting possibility of their potential use in prophylaxis or treatment against COVID-19. To understand why sulfasalazine and proguanil are effective against SARS-CoV-2 infection but others functioning in the same pathway were not (Fig. 4A), we looked more closely at the targets of each drug. Figure 5 shows that SARS-CoV-2 Orf8 binds to γ-glutamyl hydrolase (GGH) and regulates the synthesis of NO, which is necessary for viral synthesis. An additional auxiliary pathway, mediating the synthesis of NADP, can also affect NO production, although indirectly. Sulfasalazine and proguanil impinge on both of these pathways: Sulfasalazine targets the NF-κB inhibitors NFKBIA and IKBKB as well as CYP450 enzymes, whereas proguanil targets DHFR and CYP450 enzymes plus interacting partners (table S6). In this way, we hypothesize that these two drugs might more effectively target NO production and thus disrupt viral replication. By contrast, the three drugs that were not effective against SARS-CoV-2 infection (flucytosine, alogliptin, and ademetionine) only affect one of the two pathways. This analysis thereby highlights the possibility that targeting NO production through multiple pathways may provide a potential rationale for the efficacy of sulfasalazine and proguanil in reducing viral replication.

Safety is a particularly important consideration, since such drugs could be prescribed to any COVID-19–positive individuals who may have a broader range of underlying medical conditions and may not be hospitalized at the time of taking the drug. Sulfasalazine and proguanil have the potential to be used prophylactically or therapeutically. Both drugs are well-established and well-tolerated drugs (*35*, *36*). Sulfasalazine is already in use as an anti-inflammatory drug against autoimmune disorders. Given that this drug has antiviral activity (Fig. 4), this raises the possibility that sulfasalazine may act not only as an antiviral but also as an anti-inflammatory if used against COVID-19. Proguanil is used against malaria in combination with atovaquone. It has an excellent safety profile and is well tolerated when used as a prophylactic and in treatment (*37*).

A complementary study using large-scale compound screening in cultured cells has recently uncovered 100 molecules that have a partial effect on viral infectivity, 21 of which show a dose-dependent reduction of viral replication (*38*). This list of drugs does not overlap with ours, with only 2 of our 200 approved drugs being present in this list (and neither sulfasalazine nor proguanil being among them). The main reason for this apparent disparity is that only 10% of the 100 compounds tested by Riva *et al.* (*38*) are approved, whereas 100% of our 200 drugs are approved. Eight drugs in the study by Riva *et al.* (*38*) that were approved by the U.S. Food and Drug Administration (FDA) are acitretin, astemizole (now withdrawn), chloroquine, clofazimine, ingenol mebutate, remdesivir, tazarotene, and tretinoin. Two others are approved only in China (flumatinib mesylate) or Japan (tamibarotene). This highlights the major difference in the two studies: Our in silico studies identify potential antiviral drugs that are already approved and therefore at an advanced stage of repurposing, whereas Riva *et al.* (*38*) have identified compounds validated in African green monkey cells VeroE6, most of which are either in preclinical or phase 1 to 3 clinical trials. Gordon *et al.* (*1*) introduced 69 drugs (29 of which are approved by the FDA, 12 of which are in clinical trials, and 28 of which are preclinical compounds) that bound DIP. Among the 29 approved drugs, 9 drugs (captopril, chloroquine, daunorubicin, indomethacin, loratadine, lovastatin, metformin, mycophenolic acid, and sirolimus) overlap with our 200 identified drugs, and 8 of these are currently in clinical trials for SARS-CoV-2.

Computational studies aiming to identify candidate drugs for COVID-19 drug repurposing have used multistage analyses including network proximity measure analysis that are focused on DIP specifically and its interactomes (*39*–*41*). By contrast, our strategy has been to holistically construct the entire pathway of proteins that are significantly affected during SARS-CoV-2 infection, through uncovering of the hidden layer between the DIP and DEP. Because the DIP and DEP were identified from two recent papers (*1*, *2*) that generated proteomic data in two different cell lines (DIP in human embryonic kidney 293 cells and DEP in Caco-2), we also used four different network algorithms to systematically identify the key proteins (see Materials and Methods). Furthermore, our approach not only identified the 200 drugs but also used neural network analysis to predict the MoA of the drugs. This combination of unique approaches allowed us to short-list drugs associated with VR, which were then experimentally tested in monkey cell VeroE6 and human Calu-3 cells. However, similar to other network analysis studies, PPI networks usually lack the directionality that provides additional information about the types of interaction (i.e., activation or inhibition). It will be beneficial to analyze additional data that provide insights into this directionality (i.e., CRISPRi datasets showing patterns of up-/down-regulation) to overcome this limitation.

Our study has shed unanticipated new light on COVID-19 disease mechanisms and has generated promising drug repurposing opportunities for prophylaxis and treatment. Our data-driven unsupervised approach and biological validation have uncovered 160 approved drugs not currently in clinical trials, which can be investigated immediately for repurposing, and 2 drugs that show promise as antiviral drugs. We expect that this resource of potential drugs will facilitate and accelerate the development of therapeutics against COVID-19. Furthermore, our bespoke data-driven computational approach should be useful for a rapid response to new variants of SARS-CoV-2 and other new pathogens that could drive future pandemics and will also be applicable to other noninfectious disease areas with high unmet medical need.

## MATERIALS AND METHODS

### Directly interacting proteins and differentially expressed proteins

A total of 332 high-confidence SARS-CoV-2–human interactions were obtained from Gordon *et al.* (*1*) (table S3 from https://doi.org/10.1038/s41586-020-2286-9). A total of 332 high-confidence virus-host interactions were used as DIP. Data of proteome measurements by mass spectrometry at 6 and 24 hours after SARS-CoV-2 infection were obtained from Bojkova *et al.* (*2*) (table S2 from https://doi.org/10.1038/s41586-020-2332-7). The proteins that were significantly up- or down-regulated (two-sided, unpaired Student’s *t* test with equal variance assumed, *P* < 0.05, |log_2_FC| > 0.5) were selected.

### SIP network construction

The SIP network was constructed of all the shortest paths between DIP and DEP in a human PPI network from the STRING database (v11.0) (*5*). The main purpose of constructing the SIP network in our study was to identify COVID-19 disease–associated proteins. The STRING database was selected as the PPI database given the previous evidence that it contains more comprehensive information on diverse collections of disease-associated protein sets compared with other databases (*42*).

Only interactions with a confidence score of more than medium (0.4) were used. The 0.4 cutoff is the default setting and the medium level of confidence for PPI searches in the STRING database (*43*, *44*). This study used network algorithms to identify key proteins by investigating the whole network. Thus, the cutoff was used to construct a more comprehensive network that captures any potential interactions, and then the network analysis was conducted to systematically identify key proteins by analyzing all these possible interactions. The STRING database does not provide directional information.

All of the shortest paths between all pair proteins of DIP and DEP on the human PPI network were found using Dijkstra algorithm. For the shortest path finding, we used the Python package NetworkX (v2.2) (*45*). Networks were visualized using Gephi 0.9.2 (fig. S1) (*46*).

### Network analysis

Eigenvector centrality, degree centrality, betweenness centrality, and RWR were used to identify key proteins in SIP networks. The SIP network is represented by an adjacency matrix *A*, where *A_ij_* = 1 if there is an edge between nodes *i* and *j* or *A_ij_* = 0 otherwise. The eigenvector centrality *x_i_* was defined as

λx=xA (1)

where *x* is an eigenvector of the adjacency matrix *A* with eigenvalue λ. If λ is the largest eigenvalue of the adjacency matrix *A*, there is a unique solution *x*, and all centrality values are positive (*47*). Degree centrality of node *i* was defined as

$$C_{D}(i)=\sum_{j=1}^{N}A_{\mathrm{ij}}$$ (2)

where *N* is the number of nodes in the SIP network. Betweenness centrality of a node *i* was defined as

$$C_{B}(i)=\sum_{s,t\in V}\frac{\sigma (s,t|i)}{\sigma (s,t)}$$ (3)

where *V* is the set of nodes, σ(*s*, *t*) is the total number of shortest paths between *s* and *t*, and σ(*s*, *t*∣*i*) is the number of number of the shortest paths between *s* and *t* paths passing through node *i*. If *s* = *t*, σ(*s*, *t*) = 1, and if *i* ∈ *s*, *t*, σ(*s*, *t*∣*i*) = 0.

Eigenvector centrality was used to identify the most influential proteins in the network. If a protein is frequently interacted by other proteins, which also have high eigenvector centrality, then the protein will have high eigenvector centrality. Degree centrality was used to identify the hub proteins in the network. Betweenness centrality was used to identify the bottleneck proteins in the network. The betweenness centrality algorithm finds the number of the shortest paths that pass through the given protein among all protein pairs in the SIP network. RWR was used to see which human proteins were affected the most upon SARS-CoV-2 infection. To do this, we used 332 DIPs as the starting points of RWR. The RWR parameters were (i) a restart probability that is 0.15, (ii) a maximum iteration number that is 100, and (iii) an error tolerance of 1 × 10^−6^. We have assigned edge betweenness centrality as an edge score on the SIP network. The RWR calculated a score per protein in the SIP network that indicates how much a given protein was influenced by SARS-CoV-2 via DIP. The algorithms were implemented in the Python package NetworkX (v2.2) (*45*).

Permutation tests were performed 1000 times to identify significant proteins for each of the network centrality algorithms. In 1000 permutation tests, each test generated a random network with a preserved degree distribution of the original network, the SIP network. To generate a random network, we reconnected the edge in the SIP network and swiped the node. The random network in each permutation test therefore has at least 66% of the rewired edges. In the permutation test, we then applied the network algorithm and obtained the cumulative results of the network algorithm. These cumulative results were used to calculate the empirical *P* value of the network algorithm. We combined the four permutation test results to determine the final set of key proteins that have an empirical *P* value of ≤0.01 in either result.

### Key protein functional enrichment analysis

Key proteins of SIP network were tested for enrichment of DISEASES (*8*) and GO (GO biological process) terms. Enrichment analyses were performed using REST API of Enrichr (https://maayanlab.cloud/Enrichr/) (*48*).

### Visualization of a key network of SIP network

Key networks were built using interactions between the key proteins of the SIP network at 6 and 24 hours after infection. When visualizing the key networks, subcellular localization of key proteins and enriched pathways of hidden layer proteins was added (Fig. 2, B and C). Subcellular localization information for key proteins was found using COMPARTMENT database (*49*). Among the available datasets in the COMPARTMENT database, “knowledge channel” data with a confidence score of greater than four was used. The knowledge channel for humans is based on the annotations of UniProtKB, manually curated data. The confidence score of four is the highest confidence score of the knowledge channel and is only applicable to data with experimental results. To identify enriched functions of the hidden layer proteins, the hidden layer proteins were tested for enrichment of Reactome pathway terms. Most hidden layer proteins belonged to the pathways metabolism of RNA, cell cycle, and immune system, so we subdivided the hidden layer proteins into three subgroups for key network visualization. The visualization was carried out using Circos (*50*).

### Drug-target interactions

Approved drugs were collected from ChEMBL (*16*) and DrugBank (*17*). Drug-target interaction information was collected from DrugBank (v5.1) (*17*), STITCH (v5.0, confidence score > 0.9) (*51*), and Cheng *et al.* (*52*).

### In silico network-based proximity analysis

In silico network-based proximity analysis was conducted for key proteins from the SIP network at 6 and 24 hours. Given *K*, the set of key proteins from SIP networks, and *T*, the set of drug targets, the network proximity(Eq. 4) of *K* with the target set of *T* of each approved drug where *d*(*k*, *t*), the shortest path length between nodes *k* ∈ *K* and *t* ∈ *T* in the human PPIs (*52*), was executed. The closest distance measure was used to calculate the distance between a given drug’s targets to our key proteins in the SIP network because it showed the best performance in drug-disease pair prediction in the study of Guney *et al.* (*15*)

$$d_{c}(K,T)=\frac{1}{\left\|T\right\|}\sum_{t\in T}\text{mi}n_{k\in K}d(k,t)$$ (4)

To assess the significance of the distance between a key protein of SIP network and a drug *d*_c_(*K*, *T*), the distance was converted to *z* score based on permutation tests by using

$$z(K,T)=\frac{d(K,T)-\mu_{d(K,T)}}{\sigma_{d(K,T)}}$$ (5)

The permutation tests were repeated 1000 times, each time with two randomly selected gene sets. There are few high-degree nodes due to the scale-free network of the human PPI network. To avoid repetitive selection of the same high-degree nodes during random selection, we used a binning approach with at least 100 nodes in a bin. In the binning approach, nodes in the same bin have similar node degree to maintain node degree distribution for random selection. When we randomly select a set of genes, we performed a random selection among proteins from all bins so that the minimum node degree was less than the minimum node degree of the selected gene set and the maximum node degree was greater than the maximum node degree of the selected gene set. The corresponding *P* value was calculated on the basis of the permutation test results. Drug–to–SARS-CoV-2 associations with a *z* score of less than −2 were considered significantly proximal (*15*).

### Drug-pathway associations

To understand the MoAs for our 200 identified drugs, we conducted the Reactome pathway enrichment analysis for the target proteins of these drugs using R (v3.5.2) package, gprofiler2 (hypergeometric test, *P* value of <0.05) (*53*). Reactome pathway database (the version as of 15 May 2020) was used for pathway enrichment analysis because it is the most actively updated public database of human pathways (*54*). Pathway enrichment analysis was first performed using only the target proteins of each of the 200 drugs. However, 120 of 200 drugs did not have significantly enriched pathways because these drugs had fewer than six target proteins. To overcome this issue, “one-degree” neighbor proteins were added for those drugs targeting fewer than six proteins.

Significantly enriched biological pathways of drug targets for each of the 200 drugs were integrated, resulting in 148 key pathways. The Reactome pathway has a hierarchical structure among pathways. The lower hierarchy pathway is more specific than the higher hierarchy pathway. The parent pathway semantically includes the children pathways. In the process of integrating the enriched pathways per drug, we used the lowest possible hierarchy pathways to avoid the overlapping biological meaning among the hierarchical pathways.

On the basis of these identifications, a matrix containing F1 scores of the 200 drugs and the 148 key pathways was generated for drug-pathway association. The Reactome pathway enrichment analysis for the 200 drugs using gprofiler2 provides enrichment *P* values and precision and recall information that were used to produce the F1 scores. The meaning of precision here is the proportion of drug targets that are annotated to the pathway. The meaning of recall here is the proportion of the pathway gene set that the drug targets recover. The pathway to which the largest number of drug target proteins belong has the highest precision value. The pathway with the greatest intersection of pathway proteins and target proteins has the highest recall value. In other words, the pathway with the highest F1 score in the drug-pathway associations is the pathway to which the drug’s target protein belongs the most and the pathway with the largest intersection between the target proteins. For example, the number of target proteins for sulfasalazine is 13. The number of “arachidonic acid metabolism” pathway proteins is 59. The number of intersections between the target protein of sulfasalazine and the arachidonic acid metabolism pathway protein is 4. So, the precision is 4/13 = 0.3077, and the recall value is 4/59 = 0.0678. Thus, the F1 score is 0.1111. The number of “fatty acid metabolism” pathway proteins is 177, and the number of intersections between the target protein of sulfasalazine and the fatty acid metabolism pathway protein is 4. The precision is the same as 0.3077 for arachidonic acid metabolism, but the recall value is 4/177 = 0.0225. Thus, the F1 score is 0.0421, which is lower than the arachidonic acid metabolism. Hence, the F1 score complements the imbalance between the pathway protein and the target protein. This matrix was constructed using the F1 score [F1 = 2(precision × recall)/(precision + recall)] from the pathway enrichment analysis (table S8).

### MoA analysis

We used SOM (*55*) to cluster pathways based on their protein components and F1 score profiles. SOM has a descriptive ability and hence advantages in visual concept detection. Thus, it was useful to directly compare the SOM component heatmaps of the 148 pathways. SOM also has the advantage of dimensional reduction to allow a more appropriate clustering result. SOM was used followed by k-means clustering to calculate the low-dimensional abstractions that are then clustered using k-means. This two-phase approach increases the efficiency of k-means clustering with a relatively small number of samples that is a limitation in hierarchical clustering algorithms. Another advantage of SOM is noise reduction because SOM abstractions are less sensitive to random variations than the input data. In addition, SOM offers a systematic arrangement of the 200 drugs to each neuron and hence to pathway clusters (Fig. 3, D and E).

The data used in training was the F1 score matrix for drug-pathway associations (148 pathways by 200 drugs; table S8). From the SOM training, we generated a U-matix that represents the distance between neighboring nodes in the map. U-matrix of the trained unsupervised SOM contains the vector norms between the neighboring SOM nodes and shows data density in input space. Each subunit is colored according to distance between corresponding data vectors of neighbor units. Low-distance areas (dark blue) have high data density (clusters) (Fig. 3A). DBI (*56*) was calculated on the basis of the U-matrix to determine the optimal number of clusters. We used the DBI, a metric for within-cluster distance at various SOM parameters. Minimizing this index allowed discovery of groups of pathways with shared MoA or protein overlaps. The lowest DBI value occurred at nine clusters, and thus, we decided to separate the 148 key pathways into nine pathway clusters (fig. S7). K-means algorithm was then used to find the nine pathway clusters (Fig. 3B). The SOM component maps of 148 pathways (fig. S6) were analyzed on the basis of the clustering result (Fig. 3B) and mapped into two MoA categories based on the biological functions (Fig. 3C). The mapping result of 148 pathways to nine clusters and two MoA groups is available in table S9. The SOM model also labeled each neuron with the 200 drugs (Fig. 3, D and E). The detailed information of the labeled SOM neurons and the 200 drugs is available in table S6 (columns V and W). The SOM Toolbox package (*57*) for MATLAB was used for this analysis with default settings and parameters.

### Quantification of the most frequently targeted proteins among the 200 drugs

The frequency of drug-protein targeting was counted. Permutation tests were then performed 100 times to identify the significance threshold for the frequency of drug-protein targeting (fig. S8A). For each permutation test, the 200 drugs among all the drugs that we used for the in silico network-based proximity analysis were randomly selected. Then, the number of drugs targeting the same protein was calculated for all of the randomly selected 200 drugs. The proteins frequently targeted in the SIP network (empirical *P* value of <0.01) were then tested for enrichment of UniProt keywords (fig. S8B). Since UniProt keyword contains a mixture of information from 10 different categories, it was used for the enrichment test to detect any mechanistic differences among the 200 drugs.

### Cell culture

*Chlorocebus sabaeus* (green monkey) Vero E6 cells [Vero 76, clone E6, Vero E6, American Type Culture Collection (ATCC) CRL-1586] authenticated by ATCC and tested negative for mycoplasma contamination before commencement were maintained in a humidified atmosphere at 37°C with 5% CO_2_, in Dulbecco’s modified Eagle’s medium (DMEM) containing 10% (v/v) fetal bovine serum (FBS; Invitrogen). Calu-3 (ATCC HTB-55) human lung cells that tested negative for mycoplasma contamination before commencement were maintained in a humidified atmosphere at 37°C with 5% CO_2_ in Eagle’s minimum essential medium containing 20% (v/v) FBS. Human cell lines used were either not listed in the cross-contaminated or misidentified cell line database curated by the International Cell Line Authentication Committee or were previously verified by karyotyping.

### Viruses and infections

Infection experiments were performed under biosafety level 3 conditions. SARS-CoV-2 (strain München-1.2/2020/984) isolate was propagated in Vero E6 cells in DMEM supplemented with 2% FBS. For infection experiments in Vero E6 and Calu-3 cells, SARS-CoV-2 (strain München-1.2/2020/984) viral supernatant was used at multiplicity of infection (MOI) = 0.01 plaque-forming units per cell for 24 hours. All work involving live SARS-CoV-2 was performed at the BSL-3 facility of the Institute for Virology, University of Giessen (Germany), and was approved according to the German Act of Genetic Engineering by the local authority.

### Cell infection and drug treatment

Vero E6 and Calu-3 cells were seeded using 8 × 10^4^ cells in 24-well plates. The following day, cells were treated for 3 hours before infection with the indicated doses of ademetionine (30 μM; Selleckchem), alogliptin (10 μM; Selleckchem), flucytosine (300 μM; Selleckchem), proguanil (5 nM to 500 μM; Selleckchem), sulfasalazine (5 nM to 500 μM; Selleckchem), IFN-A (1000 U/ml), dimethyl sulfoxide (DMSO; Sigma-Aldrich), or mock and infected with SARS-CoV-2 at an MOI of 0.01 in serum-free DMEM at 37°C for 24 hours before RNA or protein lysis. Infection experiments were performed under biosafety level 3 conditions.

### Quantitative RT-PCR analysis

RNA was isolated using the RNeasy Mini Kit (Qiagen). SARS-CoV-2 replication (E-gene and N-gene RNA) and gene expression of the cytokines *CXCL3*, *IFNB1*, *and TNF-A* were quantified by reverse transcription quantitative polymerase chain reaction (RT-qPCR). For complementary DNA (cDNA) synthesis, RNA was reverse-transcribed with the SuperScript VILO cDNA Synthesis Kit (Invitrogen, 11755-050). The levels of specific RNAs were measured using the ABI 7900 real-time PCR machine and the PowerUp SYBR Green Master Mix (Applied Biosystems, 100029284) according to the manufacturer’s instructions. ΔCT values were determined relative to glyceraldehyde-3-phosphate dehydrogenase (GAPDH), and ΔΔCT values were normalized to infected DMSO-treated samples. Error bars indicate the SD of the mean from three independent biological replicates. All primer sequences are listed in Table 1 below.

**Table 1:** Gene names and primer sequences used in the study.

**Gene name**	**RT-PCR**	
	**Forward primer**	**Reverse primer**
*CXCL3*	GCCCAAACCGAAGTCATAGC	CAGTTGGTGCTCCCCTTGTT
*IFNB1*	GCGACACTGTTCGTGTTGTC	AGCCTCCCATTCAATTGCCA
*TNF-A*	GCTGCACTTTGGAGTGATCG	TCACTCGGGGTTCGAGAAGA
*GAPDH*	GTCTCCTCTGACTTCAACAGCG	ACCACCCTGTTGCTGTAGCCAA
*SARSCoV2-E*	ACAGGTACGTTAATAGTTAATAGCGT	ATATTGCAGCAGTACGCACACA
*SARSCoV2-N*	GACCCCAAAATCAGCGAAAT	TCTGGTTACTGCCAGTTGAATCTG

### Cytotoxicity cell viability assays

Cytotoxicity was performed in Vero E6 and Calu-3 cells using Neutral Red (Abcam, ab234039) and [3-[4,5-dimethylthiazol-2-yl]-2,5 diphenyl tetrazolium bromide (MTT)] (Roche) assay, respectively, according to the manufacturer’s instructions. Cytotoxicity was performed in Vero E6 and Calu-3 cells with the indicated compound dilutions and concurrent with viral replication assays. All assays were performed in biologically independent triplicates.

### Western blot analysis

A total of 8 × 10^4^ Vero E6 cells either mock-infected or infected and treated with DMSO or proguanil (50 μM) or sulfasalazine (200 μM) for 24 hours were resuspended and lysed in whole-cell 1× SDS sample buffer [4× SDS sample buffer: 143 mM tris-HCl (pH 6.8), 28.6% glycerol, 5.7% SDS, and 4.3 mM bromophenol blue]; supplemented with 2 ml of 2-mercaptoethanol, protease inhibitors (Sigma-Aldrich), and phosphatase inhibitors (Sigma-Aldrich); and boiled for 5 min at 95°C. A total of 10 to 20 μg of protein were separated on SDS–polyacrylamide gel electrophoresis gels and blotted onto polyvinylidene difluoride membranes (Millipore).

### Antibodies

Western blot experiments were performed using the following antibodies: GAPDH (Abcam, ab9484), phospho-MAPKAPK2 (Thr^334^, Cell Signaling Technology, 3007), goat anti-rabbit (Abcam, ab6721), and anti-mouse horseradish peroxidase (Cell Signaling Technology, 7076S).

### Statistical analysis

Statistical analyses performed are specified in the figure legends. Differences were considered significant for *P* values of <0.05.

## SUPPLEMENTARY MATERIALS

Supplementary material for this article is available at http://advances.sciencemag.org/cgi/content/full/7/27/eabh3032/DC1

## Appendix

View/request a protocol for this paper from *Bio-protocol*.
